# Piers are hotspots for benthic marine debris in an urbanised estuary

**DOI:** 10.1371/journal.pone.0274512

**Published:** 2022-12-28

**Authors:** Brie A. Sherow, Emma L. Johnston, Graeme F. Clark

**Affiliations:** 1 Evolution & Ecology Research Centre, University of New South Wales, Sydney, New South Wales, Australia; 2 Centre of Marine Science and Innovation, University of New South Wales, Sydney, New South Wales, Australia; University of Pavia, ITALY

## Abstract

Records of anthropogenic marine debris and the threats it poses are increasing worldwide, yet we know relatively little about the distribution of benthic debris. The seafloor is the final destination for a large proportion of debris due to the degradation and sinking of items. A more detailed understanding of debris distributions in hotspots such as urbanised estuaries can help decision makers target management and remediation activities. We selected sites frequented by fishers and boaters in Sydney Harbour, an urbanised estuary, to investigate the impacts of recreational activities on debris abundance. The aim of this study was to examine variation in macro debris (>5mm in diameter) type and abundance at two habitat types (piers and non-piers). We chose five locations at various distances from the estuary mouth. In each location SCUBA teams performed fixed transects at two sites, one under a pier and one over nearby soft-sediment habitat. Debris was recovered by the divers and brought to the surface for classification and disposal. Surveys were repeated multiple times at each location between November 2019 and February 2020, recording a total of 2803 debris items over 36 survey events. Overall, piers had more than ten times the debris abundance of soft-sediment sites, and much higher proportion of debris types related to recreational fishing. Over half of the debris items in this study were plastic (65%), and approximately 70% of the total debris was classified as related to recreational fishing. This trait was most prominent in debris at sites closest to the estuary mouth, likely reflecting increased fishing activity in this area. This study indicates that policy makers and community groups in urbanised estuaries should focus monitoring, reduction, and remediation efforts near artificial structures such as piers, and that public awareness campaigns should target the behaviour of recreational users of these structures.

## Introduction

The negative effects of marine debris are widely accepted by scientists, policy makers, and the general public. In addition to environmental concerns [1], marine debris is an issue for public health [2, 3], the economy [4], and society [5]. It is estimated that up to 14 million metric tonnes of plastic alone enters the ocean each year, resulting in roughly $13 billion in economic costs and impacts to than 800 marine and coastal species [6]. Despite broad acknowledgement of the problem of anthropogenic debris, the scarcity of detailed distribution studies in subtidal environments makes it difficult to develop targeted management solutions [7, 8]. Targeted debris surveys in areas most at risk, such as the seafloor of heavily urbanised estuaries, will improve our capacity to reduce this pervasive threat.

The total count of plastic items on the seafloor globally (excluding micro debris) is estimated to be between 71–116 billion pieces [9]. Despite the seafloor being recognised as a major sink for macro debris [10], benthic habitats are the least studied environment for marine debris [11]. Most marine debris literature is focused on easily accessible habitats such as beaches [12, 13] yet the composition of debris types recovered on beaches differs from that of subtidal benthic habitats. Less dispersive debris items that sink or become entangled are more commonly recorded in benthic habitats and likely do not travel far from their source [14].

Early studies of subtidal benthic marine debris occurred as a by-product of trawling surveys for fisheries counts [15, 16]. More recently, remotely operated vehicles (ROVs) have been used to survey sites with difficult terrain, often in the deep sea. ROVs conduct surveys using image-based systems that do not destroy habitat, yet they may miss items and rarely bring debris to the surface for identification [17–20]. Surveys conducted by SCUBA divers can be more thorough in debris collection, finding items otherwise buried or obscured [21, 22]. Scuba surveys can also be used in any shallow water habitat type, without disturbing the environment, and can bring items to the surface for classification and disposal [23].

Most scuba surveys of debris have been conducted in non-urbanised areas such as marine parks and recreational dive sites [24, 25]. However, in the Mediterranean, researchers found that benthic debris concentrations were higher in urbanised coastal areas than in the open ocean or deep-sea habitats [26]. Although hydrodynamic processes can funnel open water debris into deep sea canyons [27], debris within bays may not move far from the point of disposal [28]. In coastal zones, benthic debris is likely to originate from local activities and be comprised of denser materials that sink near the source [29]. Moreover, debris often accumulates where there are changes in flow or where debris encounters an obstacle such as an artificial structure [30].

Abandoned, lost, or discarded fishing gear is a major source of marine debris and is problematic due to its potential for entanglement and ingestion by marine life [31]. Of the surveys done by divers in urbanised areas, researchers in the Mediterranean recorded high concentrations of fishing gear in areas with recreational boating [32, 33] and in Brazil a high amount of fishing debris was recorded under a pier [34]. High concentrations of debris are also found in shipping lanes and fishing areas [35] and coastal debris is often deposited *in situ* by fishing and boating activities [24].

Sydney Harbour is one of the most species rich and habitat diverse urban estuaries in the world [36], yet we know little about the threat of anthropogenic debris within this estuary [37, 38]. The estuary is a drowned river valley where currents are influenced by tidal flows [39] and topography, which shape patterns of debris transport and distribution [40]. Persistent organic pollutants have been found in high concentrations within plastics in Sydney Harbour [41] and the presence of recreational boating and fishing suggests potential for macro debris accumulation [42]. Boat and shore-based fishing efforts occur throughout the harbour, with estimates of over 300,000 hours of fishing during a summer period [43]. Recreational boating makes up 70% of boat traffic in Sydney Harbour [44] with boat-based fishers positively associated with nearshore soft-sediment benthic habitats, particularly towards the estuary mouth [45]. Shore-based fishing occurs throughout the harbour, but especially at piers and wharves [45].

Here we aimed to answer two main questions for Sydney Harbour: (1) does the abundance and type of debris on the seabed vary between piers and adjacent soft-sediment sites in Sydney Harbour, and (2) does this distribution change with distance from the estuary mouth (or ocean). We hypothesised that, compared to soft-sediment habitats, debris under piers would be more abundant and dominated by recreational fishing sources, and that debris abundance would increase with distance from the estuary mouth.

## Material and methods

### Sampling locations

**Fig 1:**
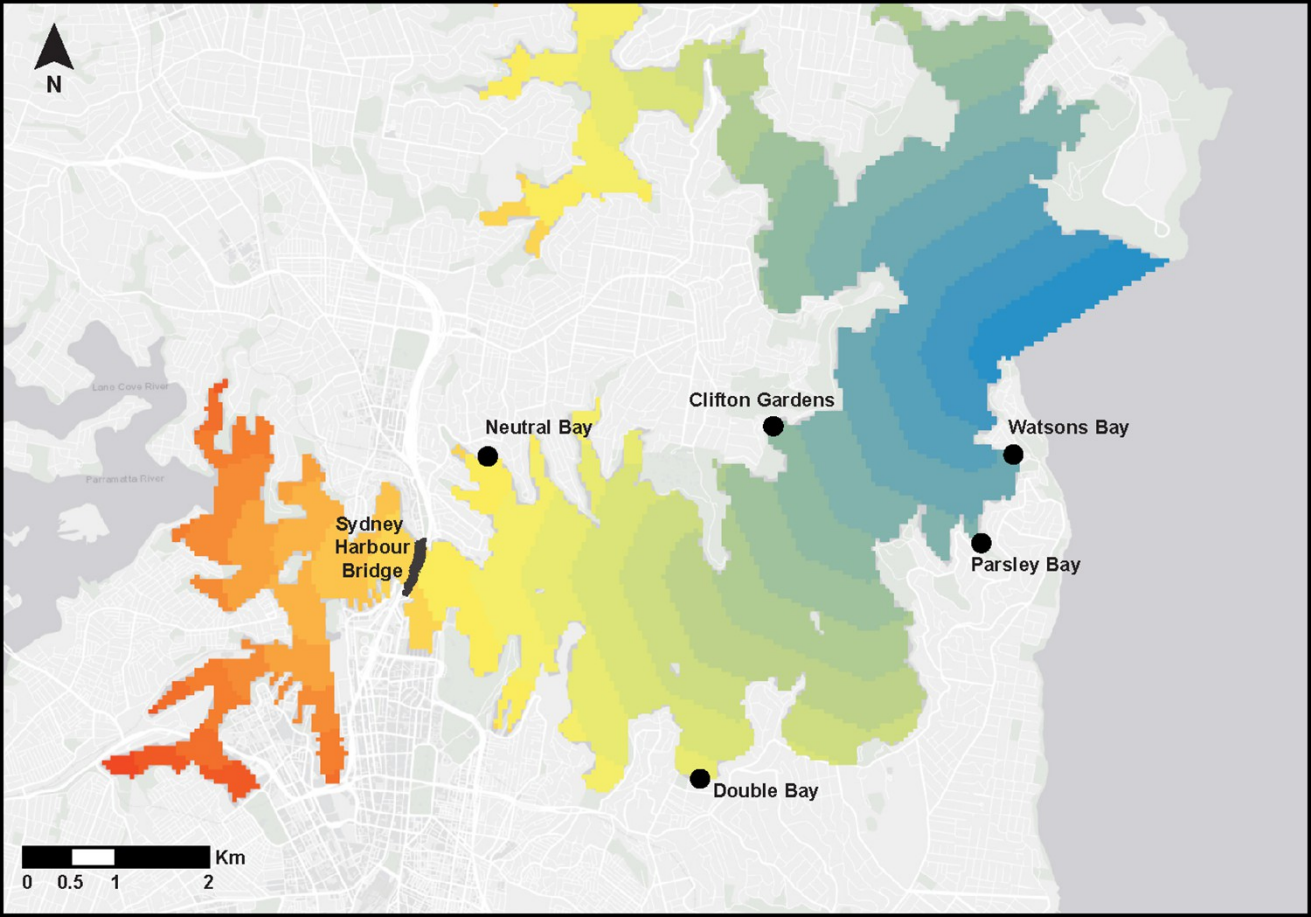
Five survey locations in Sydney Harbour. Coloured bands represent distance from the estuary mouth at 500m intervals, calculated using ‘cost distance analysis’ in ESRI ArcGIS. The location of Sydney Harbour Bridge is shown for reference.

To compare debris type and amount between pier and adjacent soft-sediment habitats we chose five locations in Sydney Harbour where both pier and soft-sediment sites were accessible to recreational users for a range of activities, at various distances from the estuary mouth (Fig 1). All locations were east of the Sydney Harbour Bridge and had both a public pier or wharf adjacent to unvegetated soft-sediment habitat used by recreational boaters. Locations included both northern and southern shores of the harbour and ranged in distance from approximately 2 km (Watsons Bay) to 9 km (Neutral Bay) from the estuary mouth. Distance from the estuary mouth was calculated using ‘cost distance analysis’ in ArcGIS [46].

### Sampling procedures

In November 2019 –February 2020, we performed 3–5 surveys at each location using modified methods from Underwater Volunteers New South Wales protocol [47]. This study was completed under permit number P13/0007-2.0 from the New South Wales Department of Planning and Environment. At each location one 25 m transect was placed on the seafloor below the pier structure and a second transect of equal length was placed across an adjacent unvegetated soft-sediment seafloor used by recreational boaters. Due to the shorter length of the pier at Parsley Bay, there the transect was placed under the full length of the pier structure (16 m) with an equivalent length transect on the adjacent soft-sediment. There was approximately 50 m between the two transects at each location, and soft-sediment transects were placed perpendicular to the end of each pier to match the average depth of the pier transects. Depths across all sites averaged approximately 3.5 m (± SD 1.4 m). Transects were composed of bricks and coloured rope secured with stakes at each end marked with subsurface buoys. These lines were left in place at each site to repeatedly survey the same area.

At each site, a pair of divers made a first pass to note conditions and record any debris that could not be removed. Both divers then started the survey together using catch bags to remove all debris within a 2.5m corridor on either side of the transect line. At the end of the transect, divers switched lanes to survey the site a second time and collect anything their dive partner had missed. The survey was completed after either two complete passes of the transect or 30 minutes had lapsed, whichever came first. Scuba teams recovered each debris item before moving along the transect, using a scuba pointer stick to uncover items that may have become entangled or buried. This technique minimised bias for items that were easy to recover versus items that were otherwise obscured. Divers cut free debris that was entangled but left non-toxic debris (such as glass) in place if it was being used by marine life for habitat.

### Debris classification

Debris was brought to the surface and then counted and classified using a modified version of the CSIRO marine debris items list [48]. CSIRO classifies debris into twelve material type categories plus an additional category for fishing related debris. We included the fishing related items in their respective material type categories and created an additional column for each debris item to record it as ‘fishing’ or ‘non-fishing’ related. We combined material categories to record ‘plastics’, ‘metal’, ‘glass’, and ‘other’. Debris categorized as ‘other’ included cloth, rubber, paper, timber, organic, e-waste, brick, and ceramic. Notes and/or photos were used to record any debris that was difficult to classify. Each piece of debris was counted individually regardless of weight or size. Enmeshed fishing line was separated and counted by piece; this method aided in recovery of items concealed inside interwoven fragments.

### Statistical analysis

In R (v4.0.3) [49], we used the package ‘glmmTMB’ (v1.2) [50] to fit generalized linear mixed models (GLMM) of debris abundance as a function of habitat (pier or soft sediment), distance from estuary mouth, and their interaction. A negative binomial distribution was chosen for debris abundance [51]. Location and date of survey were included as random effects to account for correlation between repeat surveys at the same sites. We first tested the significance of the interaction term by comparing the AIC (Akaike Information Criterion) of the full model to that of a reduced model without the interaction, using a likelihood ratio test with Chi-square distribution [52]. This was done using the ‘drop1’ function in R. If the interaction was non-significant, it was removed, and main effects were tested by the same process. We then used ‘DHARMa’ (v0.3) [53] to plot residuals and test for uniformity, outliers, dispersion, and zero inflation to verify model assumptions were met. With this approach we modelled the following response variables: 1) fishing and non-fishing related debris, and 2) debris material types (plastic, metal, glass).

We used ‘vegan’ (v2.4) [54] to create a multi-dimensional scaling (MDS) plot from a Bray-Curtis distance matrix to examine similarities between debris assemblages at each survey, and correlations between debris types survey and survey events. Environmental variables were plotted using averages of ordination scores for factor levels with ‘envfit’. Categorical environmental variables (pier and soft sediment site types) were represented by ellipses plotted using standard deviation with ‘veganCovEllipse’. A continuous environmental variable (distance to harbour mouth) was represented as an axis showing the strength of the association, with the plotted segment scaled to the r^2^ value using ‘ordiArrowMul’. We tested MDS stress and ran PERMANOVA analysis to test for difference between survey events grouped by habitat and distance from estuary mouth.

We used generalised linear latent variable models ‘gllvm’ (v1.2) [55] with fourth corner terms [56] to explore the interactions between debris traits’ response to environmental variables in the model. For this analysis, we selected two environmental variables: habitat (pier or soft sediment), and distance to estuary mouth; and three debris traits: dispersiveness of debris (very mobile, encumbered drift, and limited dispersion), material type (plastic, glass, metal, other), and whether the item was classified as fishing related. Debris abundance was regressed against debris traits and environmental variables with interactions between these variables. These interaction terms allowed us to investigate how debris response to the environment depended on their traits (e.g. fishing related). Latent variables accounted for correlations between the multivariate response variables. Plotting these latent variables revealed patterns of co-occurrence of debris that was not due to common response to variables in the model.

## Results

After 36 survey events, a total of 2803 debris items were counted at pier and soft-sediment sites at 5 locations. Over ten times the amount of debris was recovered at pier sites compared to soft-sediment sites, with an average density of 1.22 and 0.12 debris items per m^2^ transect in pier and soft-sediment sites respectively. Over half of these debris items were plastic (65%), followed by metal (17%), glass (9%), and other materials (9%). Debris items classified as fishing-related accounted for 69% of the total. The top items recovered across all surveys were primarily associated with recreational activities including fishing debris, glass beverage bottles, food wrappers, and plastic scraps. Abundant items associated with industrial activities included bricks and metal scraps.

Plastic was the most common material type recorded in both pier and soft-sediment habitats, making up 66% of total debris recovered under piers and 46% of total debris recovered on soft sediment sites. At piers, the second most abundant material type was metal (18%) while glass was the second most common material type recovered on sediment sites (16%).

**Fig 2:**
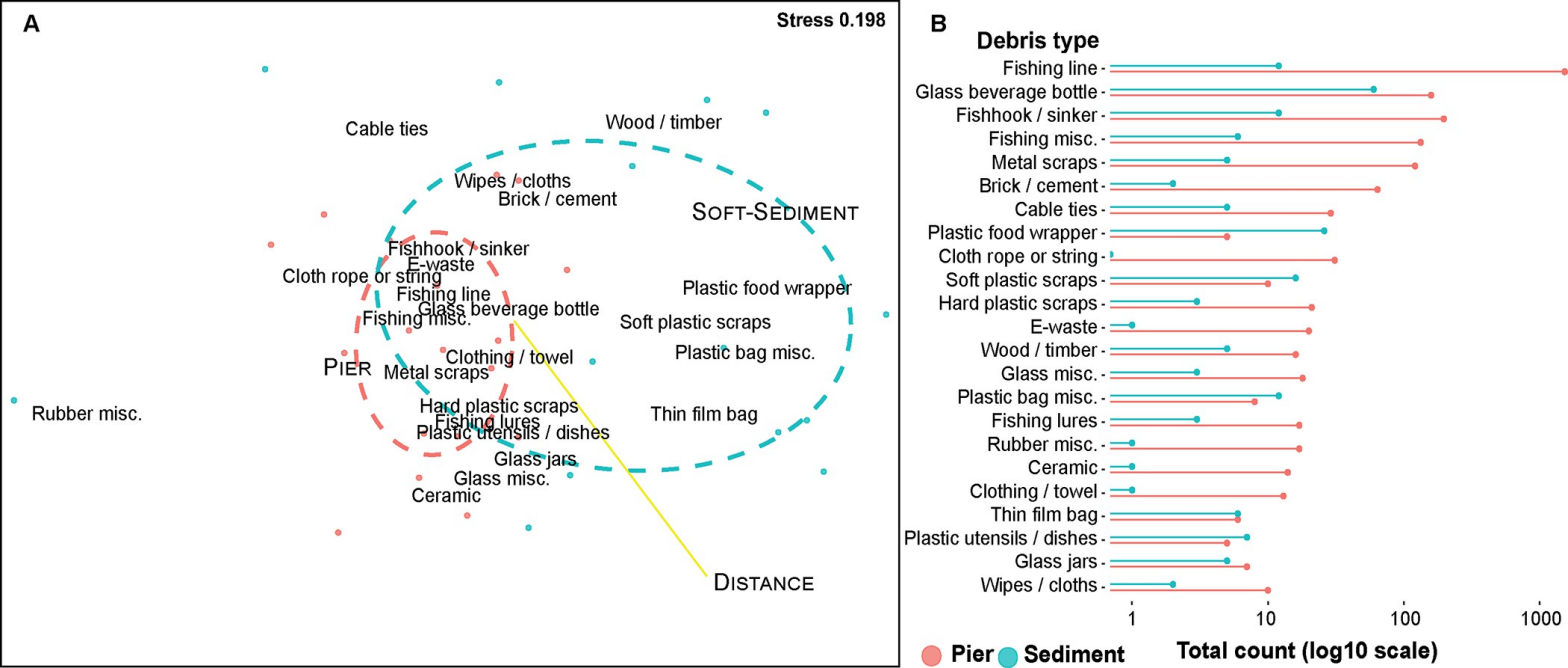
Debris item types in both pier and soft-sediment habitats. A) Multi-dimensional scaling (MDS) plot showing influence of environmental variables and similarity between survey events using ‘Bray-Curtis’ distance matrix. Red points represent pier surveys and blue points represent soft sediment surveys. Ellipses were calculated using standard deviation with ‘veganCovEllipse’, with red representing pier surveys and blue representing soft sediment surveys. Fit of distance to harbour mouth represents the strength of the association, with a longer segment more strongly correlated to the data. B) Total item count of abundant debris types per pier or soft-sediment habitat. Debris count was plotted with a log 10 scale.

Survey events at piers were more tightly clustered in MDS plot than were soft-sediment surveys, showing greater similarity in debris types and abundance (Fig 2A). Fishing related items were tightly clustered and were correlated with clothing, e-waste, and plastic cutlery. Debris items showing the least similarity between surveys included treated timber, and rubber scraps. Plastic fishing line was the dominant debris item overall, representing 56% of items recovered across both habitat types (Fig 2B). Other items abundant in both habitats included glass beverage bottles and metal fishhooks/sinkers. There were notable differences between the most abundant debris items in the two habitat types. Items commonly abundant in pier habitats included metal scraps, bricks, and miscellaneous fishing items. Almost all debris types were more abundant in pier habitats; the exceptions being soft plastic scraps, plastic bags, plastic utensils, and plastic food wrappers which were more common in soft-sediment sites (Fig 2B). Difference in debris abundance between pier and sediment sites decreased at sites further from the estuary mouth (Fig 2A, PERMANOVA df = 1, F = 5.59, p = 0.001).

**Fig 3:**
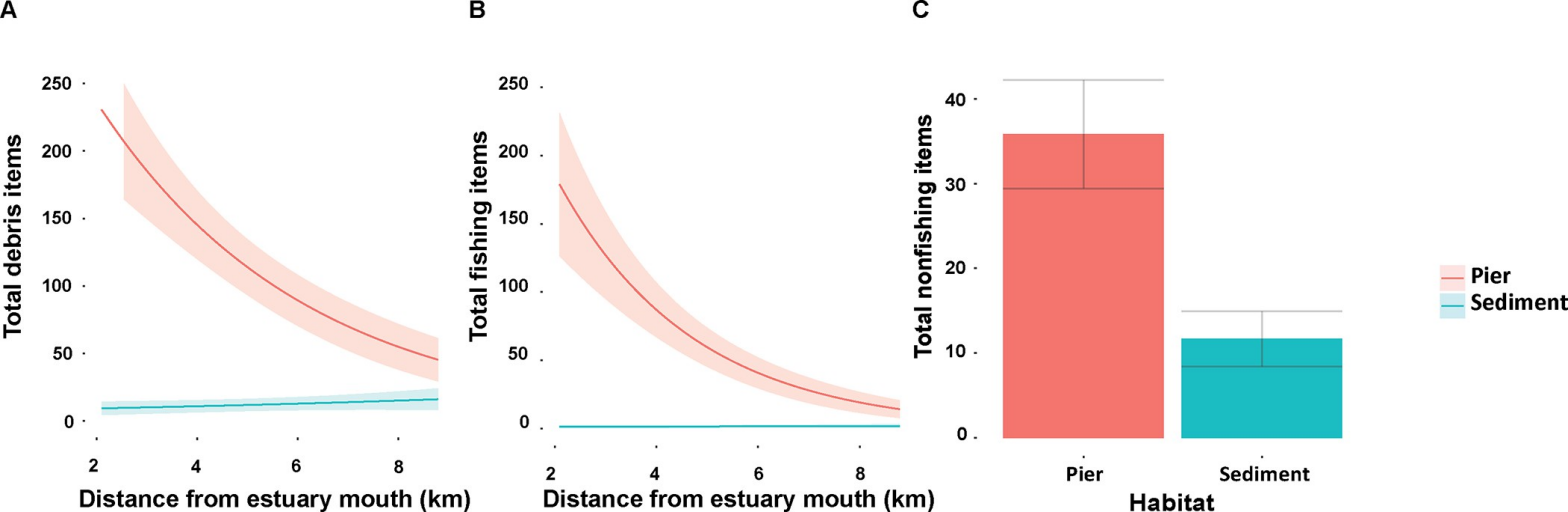
Debris abundance. Predicted means and standard errors from generalised linear mixed model (GLMM) examining the abundance of (A) total debris items, (B) total debris items classified as fishing-related, and (C) total debris items classified as non-fishing related. Shaded areas and error-bars represent standard error at 95% confidence interval.

There was strong evidence that total debris abundance was highest at pier sites near the estuary mouth Table 1. At pier habitats, total debris decreased sharply with distance from the estuary mouth, while at sediment habitats debris increased slightly (Fig 3A). Fishing related debris was more abundant under piers nearer the estuary mouth (Fig 3B) and sharply decreased with distance, while non-fishing related debris abundance was correlated with pier habitats regardless of distance from estuary mouth (Fig 3C).

**Table 1:** Model selection values.

Model Terms	AIC	LRT	p-value	AIC	LRT	p-value	AIC	LRT	p-value
	**Total Debris**			**Fishing related**			**Non-fishing related**		
Habitat	--	--	--	--	--	--	309.46	13.49	<0.001*
Distance	--	--	--	--	--	--	295.98	0.01	0.904
Interaction	345.58	7.67	0.006*	257.93	6.62	0.01*	297.97	0.70	0.402
	**Plastic**			**Metal**			**Glass**		
Habitat	--	--	--	262.45	31.27	<0.001*	--	--	--
Distance	--	--	--	232.33	1.15	0.284	--	--	--
Interaction	304.81	22.8	<0.001*	233.18	3.07	0.08	201.49	5.6	0.018*

AIC refers to the AIC of the model when term is dropped from the model, with lower AIC indicating a superior model. Likelihood ratio test (LRT) assesses the model’s goodness of fit using chi-squared distribution, and p-value represents rejection of null hypothesis (starred as significant at <0.05). Distance refers to distance from the estuary mouth. Terms were not reported individually where the interaction was found to be significant using a likelihood ratio test with Chi-square distribution.

**Fig 4:**
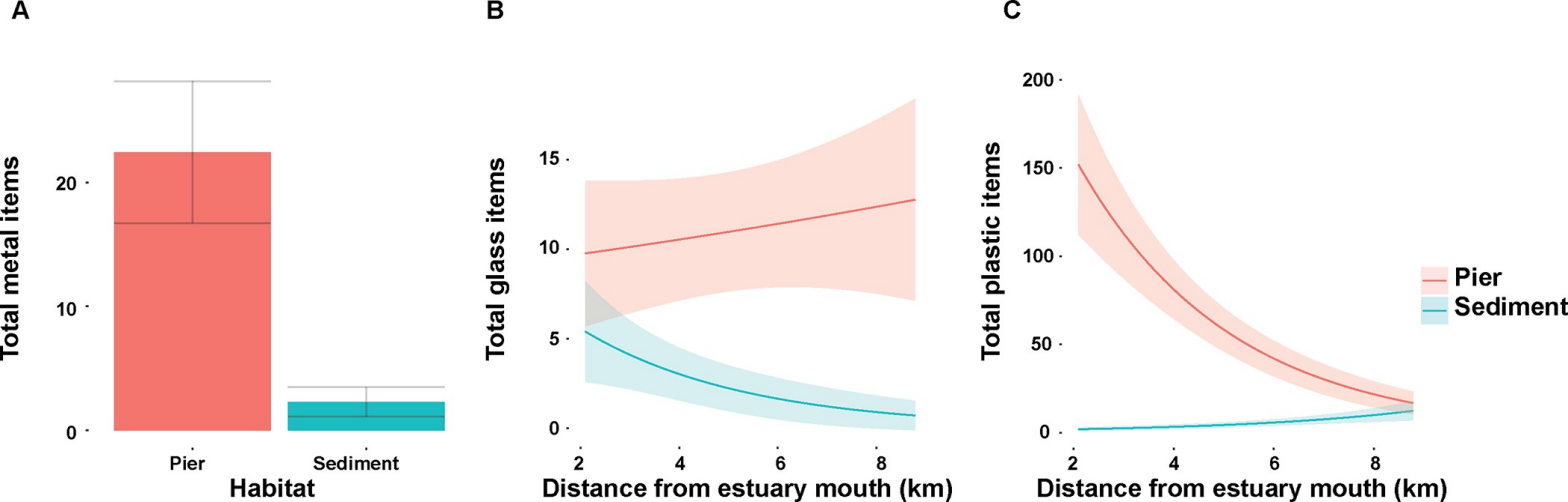
Debris abundance by material type. Predicted mean and SE from generalised linear mixed models (GLMM) examining the abundance of A) metal debris, B) glass debris, and C) plastic debris. Shaded areas and error-bars represent standard error at 95% confidence interval.

Glass and metal debris were both more abundant under piers than at soft-sediment sites, and glass debris increased at piers and decreased at sediment sites with distance from the estuary mouth (Fig 4A and 4B). Plastic debris was more abundant at pier habitats nearer the estuary mouth, while at soft-sediment habitats it was more abundant further from the estuary mouth (Fig 4C). Predictions of the abundance of plastic debris become similar at pier and sediment habitat types at approximately 10 km from the harbour mouth. All debris material types were more abundant under piers than on soft-sediment habitats, however glass debris showed the least difference in abundance between habitats Table 2.

**Table 2:** Summary of generalised linear mixed model (GLMM) estimates and test statistics.

Model Terms	Est	SE	z-value	p-value	Est	SE	z-value	p-value	Est	SE	z-value	p-value
	**Total debris**				**Fishing**				**Non-Fishing**			
Intercept (Pier)	5.98	0.43	13.91	<0.001*	5.98	0.43	13.91	<0.001*	3.58	0.18	20.05	<0.001*
Habitat (Soft- Sediment)	-5.82	0.91	-6.40	<0.001*	-5.82	0.91	-6.40	<0.001*	-1.12	0.30	-3.75	<0.001*
Distance	-0.38	0.09	-4.39	<0.001*	-0.38	0.09	-4.39	<0.001*	--	--	--	--
Habitat:Distance	0.43	0.15	2.85	0.004*	0.43	0.15	2.85	0.004*	--	--	--	--
	**Plastic**				**Glass**				**Metal**			
Intercept (Pier)	5.71	0.37	15.26	<0.001*	2.19	0.57	3.88	<0.001*	3.11	0.25	12.21	<0.001*
Habitat (Soft Sediment)	-5.6	0.66	-8.49	<0.001*	0.13	0.94	0.14	0.889	-2.28	0.44	-5.22	<0.001*
Distance	-0.33	0.07	-4.52	<0.001*	0.04	0.09	0.44	0.660	--	--	--	--
Habitat:Distance	0.60	0.10	5.96	<0.001*	-0.35	0.22	-1.59	0.111	--	--	--	--

Shown are the estimates, standard error (SE) at 95% confidence interval, z-value (distance from the mean in standard deviations) and p-value (starred at <0.05 significance).

**Fig 5:**
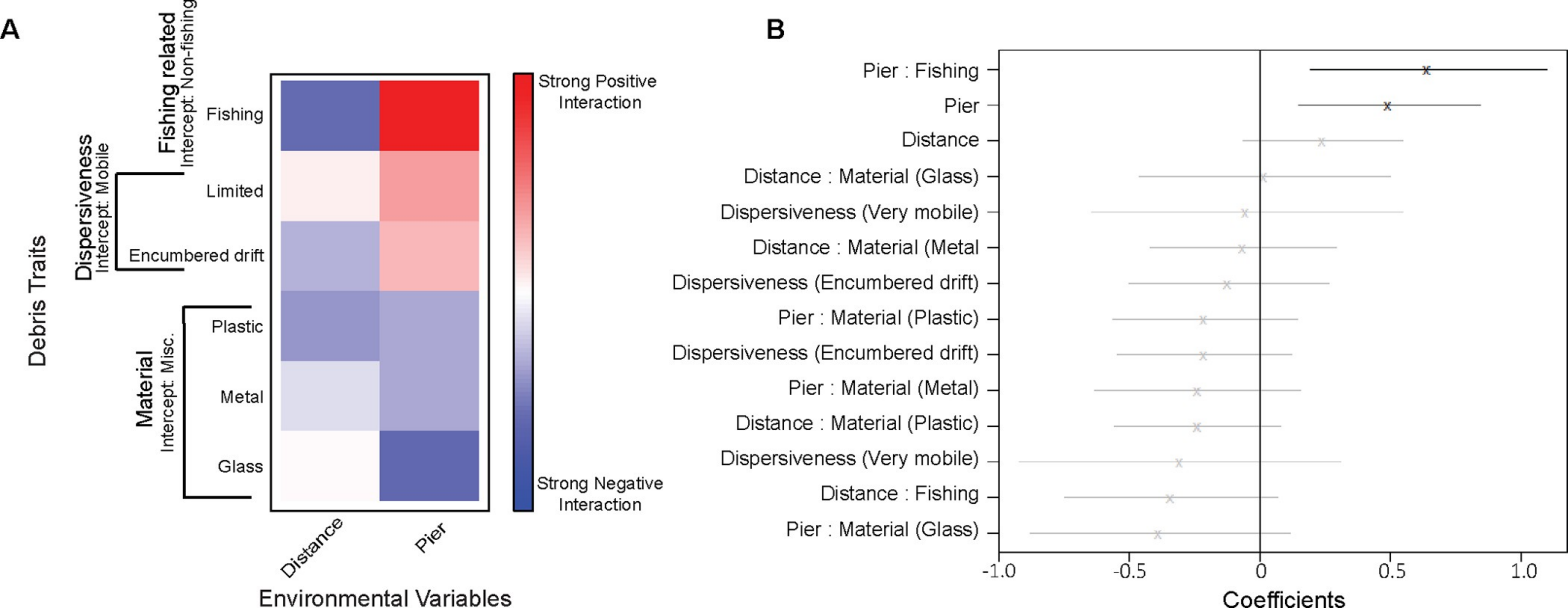
General linear latent variable model (gllvm) results. A) Interactions between environmental variables and debris traits. Darker red indicates substantial positive interaction and darker blue indicates substantial negative interaction (E.g. fishing debris is strongly associated with increased abundance at pier habitats compared to non-fishing related debris). B) Plot of environment by debris trait interaction coefficients estimates marked by x and 95% confidence intervals marked by lines. Grey confidence interval lines include 0 while black lines were significant.

Items classified as fishing-related had a strong positive association with pier habitats compared to items classified as non-fishing, and a strong negative association with distance from the estuary mouth (Fig 5A). Plastic items had a stronger negative association to distance than metal debris, while glass debris had a weak positive association with distance in comparison to the intercept. Glass debris had a stronger negative association to pier habitats than either plastic or metal. The dispersiveness of debris had weak associations with both distance and habitat (Fig 5A). We did not see significant evidence of a detectable association for debris traits of dispersiveness on abundance (Fig 5B).

## Discussion

This study was the first to document patterns of debris type and abundance across habitats and distance in Sydney Harbour. We found that the concentrations of marine debris on the seafloor under piers was ten-fold that at adjacent soft-sediment habitats, and that debris abundance increased nearer the estuary mouth. Approximately 70% of total debris items recorded in this study were classified as fishing-related, and intuitively, boat-based fishing was concentrated at near shore bays towards the estuary mouth and at piers throughout the harbour [45]. These results indicate a need for more effective management of anthropogenic benthic marine debris in highly urbanised areas, particularly in areas that are commonly used by recreational fishers.

Debris abundance at pier and sediment sites became more similar with increasing distance from the estuary mouth. This may be due to the spatial distribution of recreational fishing, since an estimated 60% of total fishing activity occurs east of the Sydney Harbour Bridge, nearer the estuary mouth [43]. Another driving factor may be weaker currents in soft-sediment sites further from the estuary mouth that allow debris to accumulate more readily. Sediment sites closer to the estuary mouth may be more strongly impacted by wind and waves which resuspend or promote the dispersion of debris more readily without the interference of a pier structure. Glass was the only material type to suggest significant increase at piers compared to sediment habitats with distance from the estuary mouth, possibly influenced by use of the sites or the nature of glass to sink immediately whereas other debris types may move through the water to deeper channels or the open ocean in sites closer to the estuary mouth [57], particularly in an estuary such as Sydney Harbour that is highly influenced by tidal flows [39]. Although dispersiveness did not have a detectable influence as a debris trait in this study, further research is needed to understand the ways in which various debris types move through the marine environment and where they settle in a final destination [58].

In addition to fishing-related debris, this study recorded considerable amounts of non-fishing related debris items such as bricks and metal scraps. This indicates that ease of access may be a factor influencing debris abundance around piers, which can serve as a convenient dumping ground [24]. Another factor influencing subtidal debris at pier sites is the increased structural complexity provided by piers, which increases the capture and retention of debris [59]. The underwater structures reduce the speed of currents, prompting debris items within the water column to settle on to the seabed [60]. The structures further provide the opportunity for entanglement (e.g. fishing line), which will accumulate over time as more debris is entrapped [61].

In comparison, soft-sediment habitats lack structural features that provide opportunities for entanglement or promoting the settling of floating debris. However, debris counts from soft-sediment sites in Sydney Harbour were proportionately higher than comparative studies in open ocean marine parks. In the South Atlantic Bight one study found ~2% of total debris surveyed on sandy bottom sites as opposed to more complex topography [62], compared to 10% on soft-sediment sites in this study. Furthermore, debris densities averaged across site types in this urbanised estuary were ~130 times higher than averages across site types in an open ocean marine park; 67 items (extrapolated) and 0.52 items per 100 m^2^ transect, respectively [63]. A study in Portugal found 1.3–17.3 macroplastic items per 100 m^2^ in saltmarsh and seagrass habitat [64] in comparison to ~12 (extrapolated) in un-vegetated soft sediment in this study.

The hydrological processes in Sydney Harbour vary with distance from the estuary mouth [65], and further research is needed to explore the influence of geological and hydrological factors on debris movements between site types within the estuary. For example, deeper channels in the centre of the estuary and sites west of the Sydney Harbour Bridge have weaker flows will likely have distinct debris profiles. Considering the high concentrations of debris under piers, the trends near other artificial structures (e.g. breakwaters) should also be explored. Benthic habitats such as rocky reefs and seagrass meadows will each have difference characteristics effecting debris concentration [66, 67]. Further research is needed to understand debris accumulation at different types of complex urbanised structures, as well as the impact of seasonal differences or events such as public holidays and storms. While this study focused on macro debris (>5 mm), the nature of debris to fragment near its origin [68] suggests a need for further research to determine the presence of fragmented debris in different seafloor types.

Globally, plastic items are projected to account for between 50–90% of total marine debris [69]. The prevalence of plastic debris in this study (65% of the total items) was higher than in comparable surveys in coastal New South Wales, Australia (33%) [24] and the Mediterranean (~55%) [22, 26]. The total amount of fishing related items in global marine debris is estimated to be 10%, yet fishing-related debris abundance is highly variable [70]. It is estimated that 29% of fishing lines are lost annually [31], and fishing-related debris is particularly concerning due to the navigational and ecological hazards posed by the items [71]. The proportion of debris classified as fishing-related in this study was higher than that reported along coastal New South Wales, Australia (38%) [24] and the Great Barrier Reef (17%) [63], although lower than along the urbanised coast of Queensland, Australia (79%) [63] and under a coastal jetty in Brazil (98%) [34].

Many of the top item types recovered in this study, primarily plastics, have well-researched negative ecological impacts. Examples include ghost fishing [72], entanglement [73, 74], ingestion [1, 75], ecotoxicology [3], introduction of invasive species [76], susceptibility to disease for impacted organisms [77], and suffocation to benthic inhabitants [78]. These examples are often given at the individual level, but when amassed, may lead to wider ecological harm, particularly when combined with other anthropogenic stressors [1, 69].

To effectively manage debris, plans should prioritise at-source reduction and consider both environmental and social processes that drive debris abundance [79]. Public awareness campaigns can target the behaviour of recreational users and reduce the amount of debris deposited *in situ* [80]. Prevalence of items classified as fishing-related suggest that outreach directed at recreational fishing communities may lead to reduced benthic debris. For example, in Australia, the citizen science initiative Tangaroa Blue created a campaign to address fishing litter [81] and OceanWatch Australia developed specialised bins to collect fishing debris, collecting over 10 tons of monofilament line in an eight year period [82]. Partnerships between researchers, engaged citizens, and policy makers can lead to meaningful change [83], and results of our study indicate that in-water remediation efforts could be focused near artificial structures such as piers both for ease of access and to target at-source debris concentrations, particularly at sites near the estuary mouth.

## Conclusion

This study demonstrates that piers in urbanised estuaries can have an order of magnitude (~10x) higher concentrations of benthic subtidal debris than nearby soft sediment habitats and should therefore be targets of reduction and removal campaigns. Debris classified as fishing-related was highly abundant at piers, particularly in the outer harbour, suggesting that management actions targeted at recreational fishers may be the most effective. Examples of such management actions include installation and signposting of monofilament line bins, and public awareness campaigns of the negative impacts of fishing related debris. Results of this study can provide baseline data to test the effectiveness of management plans, and remediation efforts reinforced by regular monitoring can mitigate the threats posed by benthic marine debris at piers.
